# Galectin-9 Is a Possible Promoter of Immunopathology in Rheumatoid Arthritis by Activation of Peptidyl Arginine Deiminase 4 (PAD-4) in Granulocytes

**DOI:** 10.3390/ijms20164046

**Published:** 2019-08-19

**Authors:** Valerie R. Wiersma, Alex Clarke, Simon D. Pouwels, Elizabeth Perry, Trefa M. Abdullah, Clive Kelly, Anthony De Soyza, David Hutchinson, Paul Eggleton, Edwin Bremer

**Affiliations:** 1 Department of Hematology, University Medical Center Groningen, University of Groningen, 9713GZ Groningen, The Netherlands; 2 University of Exeter Medical School, Exeter EX1 2LU, UK; 3 Department of Pathology and Medical Biology, University of Groningen, University Medical Center Groningen (UMCG), 9713 GZ Groningen, The Netherlands; 4 Department of Rheumatology, University Hospitals Bristol NHS Foundation Trust, Bristol BS1 3NU, UK; 5 Rheumatology Department, Queen Elizabeth Hospital, Gateshead NE9 6SX, UK; 6 Institute for Cellular Medicine, Newcastle University & Sir William Leech Centre, The Freeman Hospital, Newcastle NE2 4HH, UK; 7 Department of Rheumatology, Royal Cornwall Hospital Trust, Truro TR1 3UT, UK; 8 UCB Celltech, Immunobone Therapeutic Area, Slough SL1 3WE, UK

**Keywords:** rheumatoid arthritis, bronchiectasis, neutrophil, granulocytes, citrullination, inflammatory cytokines, peptidyl-arginine deiminase, galectin

## Abstract

The aetiology of rheumatoid arthritis (RA) is unknown, but citrullination of proteins is thought to be an initiating event. In addition, it is increasingly evident that the lung can be a potential site for the generation of autoimmune triggers before the development of joint disease. Here, we identified that serum levels of galectin-9 (Gal-9), a pleiotropic immunomodulatory protein, are elevated in RA patients, and are even further increased in patients with comorbid bronchiectasis, a lung disease caused by chronic inflammation. The serum concentrations of Gal-9 correlate with C-reactive protein levels and DAS-28 score. Gal-9 activated polymorphonuclear leukocytes (granulocytes) in vitro, which was characterized by increased cytokine secretion, migration, and survival. Further, granulocytes treated with Gal-9 upregulated expression of peptidyl arginine deiminase 4 (PAD-4), a key enzyme required for RA-associated citrullination of proteins. Correspondingly, treatment with Gal-9 triggered citrullination of intracellular granulocyte proteins that are known contributors to RA pathogenesis (i.e., myeloperoxidase, alpha-enolase, MMP-9, lactoferrin). In conclusion, this study identifies for the first time an immunomodulatory protein, Gal-9, that triggers activation of granulocytes leading to increased PAD-4 expression and generation of citrullinated autoantigens. This pathway may represent a potentially important mechanism for development of RA.

## 1. Introduction

The galectin family of carbohydrate binding proteins, of which nine different members are expressed in humans, have various functions in the human body [1]. Within this family, Galectin-9 (Gal-9) has gained attention as a regulator of cell adhesion and polarity, induction of cancer cell death, and regulator of both adaptive and innate immunity [2,3,4]. In respect of its immunomodulatory functions, Gal-9 was initially predominantly described as a negative regulator of T cell immunity by inducing apoptotic cell death in CD4^+^ T helper 1 (Th1) and T helper 17 cells [5,6], whereas Gal-9 had a stimulatory effect on regulatory T cells (Treg) [6,7]. Autoimmune diseases are generally characterized by over activated T cell responses, showing a dominant pathogenic Th1 and Th17 phenotype and impaired Treg activity [8]. Correspondingly, treatment with Gal-9 at high doses ameliorated the severity of inflammation in various mouse models of autoimmune diseases [6,7,9,10,11]. Of note, we and others have previously demonstrated that treatment of resting mononuclear cells with lower concentrations of Gal-9 activates and expands IFN-γ producing CD4^+^ Th1 cells [12,13], suggesting that at physiological concentrations Gal-9 may contribute to immune-pathology. 

In addition to T cell immune responses, the polymorphonuclear leukocytes (granulocytes) have also emerged as important regulators of autoimmunity [14], particularly in rheumatoid arthritis (RA) [15]. RA is a chronic inflammatory disease that mainly affects the joints of which the exact etiology is currently unknown. However, citrullination of proteins by protein-arginine deiminases (PADs) is thought to be an initiating event. Indeed, the presence of anti-citrullinated protein antibodies (ACPAs) in the serum is considered a hallmark of RA [16] and can already be detected years before clinical manifestation of the disease [17,18]. The contribution of granulocytes to the immunopathology of RA has been shown in mouse models where depletion of granulocytes completely prevented development and progression of RA [17,18,19,20]. Further, circulating granulocytes of RA patients are more primed for the production of reactive oxygen species (ROS) [21] and show enhanced NETosis [22] compared to healthy controls. Such activated granulocytes could be a source of citrullinated autoantigens. However, the mechanism by which granulocytes are activated in RA have yet to be completely elucidated.

Interestingly, Gal-9 has been described as a modulator of granulocyte activity, mainly in the context of lung inflammation. For instance, Gal-9 induced the degranulation of granulocytes and primed them for enhanced NADPHD oxidase activity, which contributed to the killing of *Pseudomonas aeruginosa* [23]. Similarly, Gal-9 increased in vitro ROS production by granulocytes in the context of *Francisella novicida* infection and infiltration of CD11b^+^Ly6G^+^ granulocytes upon infection was reduced in Gal-9 knock-out mice [24]. In line with the granulocyte stimulating activity of Gal-9, the concentration of Gal-9 in bronchoalveolar lavage fluid (BALF) and its expression in lung tissue was found to be elevated during lung infection [24,25]. 

It is increasingly evident that the lung can be a potential site for the generation of autoimmune triggers before the development of joint disease [26,27,28,29]. Especially, smoking [30,31,32,33] and the lung disease bronchiectasis (BR), a complex and heterogeneous chronic lung disease in which foreign material and bacteria in the airway trigger a vicious and recurrent cycle of excessive host-mediated granulocyte inflammation, is a risk factor for developing RA [34,35,36,37]. As Gal-9 can activate granulocytes, Gal-9 may possibly drive granulocyte mediated inflammation that leads to the initiation and progression of RA. Of note, galectins have been implicated as contributors to RA pathogenesis [38].

In the present study, we determined serum Gal-9 levels in RA patients with and without bronchiectasis and analyzed the in vitro effects of Gal-9 on granulocyte and PAD-4 activity. These data suggest that Gal-9 possibly plays a role in granulocyte-driven inflammation in RA and may represent a causative link between BR and RA development. 

## 2. Results

### 2.1. Gal-9 Is Elevated in Serum of RA Patients and Correlates with Certain Clinical Parameters

**Figure 1:**
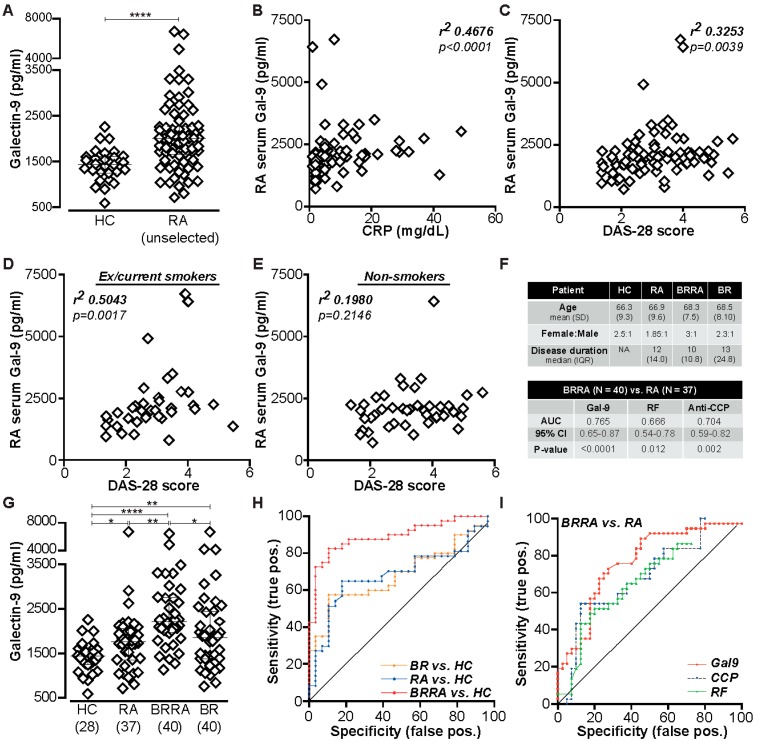
Galectin-9 serum levels in rheumatoid arthritis (RA), bronchiectasis (BR), and BRRA patients with and without BR and association with disease activity. Galectin-9 (Gal-9) concentrations were measured by enzyme linked immunosorbent assay (ELISA) in serum from (**A**) healthy controls (HC) subjects (*n* = 28) and unselected RA (*n* = 77), showing median ± IQR of 1437 ± 1007 vs. 2012 ± 621 pg/mL (*p* < 0.0001). (**B**) Correlation of Gal-9 in unselected RA patients (*n* = 77) with CRP levels. (**C**) Correlation of Gal-9 in unselected RA patients (*n* = 77) with DAS-28 scores. (**D**) Correlation of Gal-9 levels and DAS-28 in RA ex/current smokers. (**E**) Correlation of Gal-9 levels and DAS-28 in RA non-smokers. (**F**) Demographics of patients and control groups used in this study. (**G**) Gal-9 levels in HC (*n* = 28; median ± IQR 1437 ± 1007) compared with RA (*n* = 37; 1762 ± 707), BRRA (*n* = 40; 2213 ± 779), and BR alone (*n* = 40; 1847 ± 1065). (**H**) Receiver operating characteristic (ROC) curves of Gal-9 diagnostic utility in BRRA (AUC = 0.89; *p* < 0.0001; 95% CI 0.82–0.97), RA (AUC = 0.69; *p* = 0.0102; 95% CI 0.56–0.82) and BR patients (AUC = 0.69; *p* = 0.0083; 95% CI 0.56–0.81). (**I**) ROC curves for the diagnostic utility of Gal-9, antibodies to cyclical citrullinated peptide (anti-CCP), and rheumatoid factor (RF) in BRRA vs. RA patients. The area under the curve for Gal-9 in the ROC curve was 0.77 (*p* < 0.0001), for anti-CCP 0.70 (*p* < 0.0021; 95% CI 0.59–0.82), and RF 0.67 (*p* < 0.0121; 95% CI 0.54–0.79). *p* values * = 0.05; ** = 0.01; **** = 0.0001.

Since we hypothesized that Gal-9 may contribute to autoimmune pathology in RA, we analyzed its serum concentration in RA patients and age/sex-matched healthy controls (HC). Gal-9 levels were significantly elevated in RA compared to HC (Figure 1A) and significantly correlated with CRP levels (Figure 1B) and DAS-28 score (Figure 1C). Of note, Gal-9 levels strongly correlated with DAS-28 scores in 36 RA patients that were ex or current smokers (Figure 1D) but did not correlate with DAS-28 in non-smokers (Figure 1E). No obvious statistically significant correlation between Gal-9 levels and DAS-28 disease activity was observed in non-smokers. This may be explained in part by reduced Gal-9 levels in both the BRRA and RA non-smoking groups compared to their matched disease smoking samples. As shown in Figure S1A, the median Gal-9 levels of BRRA and RA patients who smoked was 2286 and 1908 pg/mL, respectively, which showed a statistically significant positive correlation with DAS-28 (r^2^ 0.5043; *p* = 0.0017) as shown Figure 1D. 

Based on this association with smoking and the fact that Gal-9 is implicated in lung pathology, we next separately evaluated RA patients, BR patients, and patients with both BR and RA (BRRA) and compared them with subjects with no autoimmune disease. The patients and controls were well matched for age and sex (see patient characteristics in Figure 1F) and patients with BR showed typical pathological changes in the lung (Figure S2A). Interestingly, BRRA patients had significantly elevated serum Gal-9 levels compared to RA and BR patients (Figure 1G). Next, we analyzed the association of Gal-9 levels in RA and BRRA patients and antibodies to cyclical citrullinated peptide (anti-CCP), but did not observe a direct positive correlation. However, BRRA patients had higher median Gal-9 serum levels irrespective of smoking history compared to RA patients without lung disease (Supplementary Figure S1A). Moreover, a greater number of BRRA patients (36/40—90%) were positive for anti-CCP than RA patients (23/35—65.7%) and had higher levels of anti-CCP antibodies (Figure S1B). Indeed, the threshold of our anti-CCP clinical assay had an upper detection limit of 600 U/mL and 20% (8/40) BBRA patients had anti-CCP levels at the upper limit of testing. In contrast, none of the 35 RA patients had anti-CCP levels of 400 U/mL or above. Hence, high anti-CCP levels were more often found in BRRA patients, and this subpopulation of RA patients in general also has higher Gal-9 levels. Receiver operating characteristic (ROC) curves of serum Gal-9 revealed a high sensitivity and specificity of serum Gal-9 for BRRA compared to HC (Figure 1H), with ROC curves for RA and BR alone being less predictive. Moreover, the ROC curve for Gal-9 had a higher diagnostic capability to distinguish BRRA vs. RA than anti-cyclic citrullinated antibodies (anti-CCP) or rheumatoid factor titer (Figure 1I).

### 2.2. Gal-9 Induces CRD-Dependent Activation of Granulocytes

**Figure 2:**
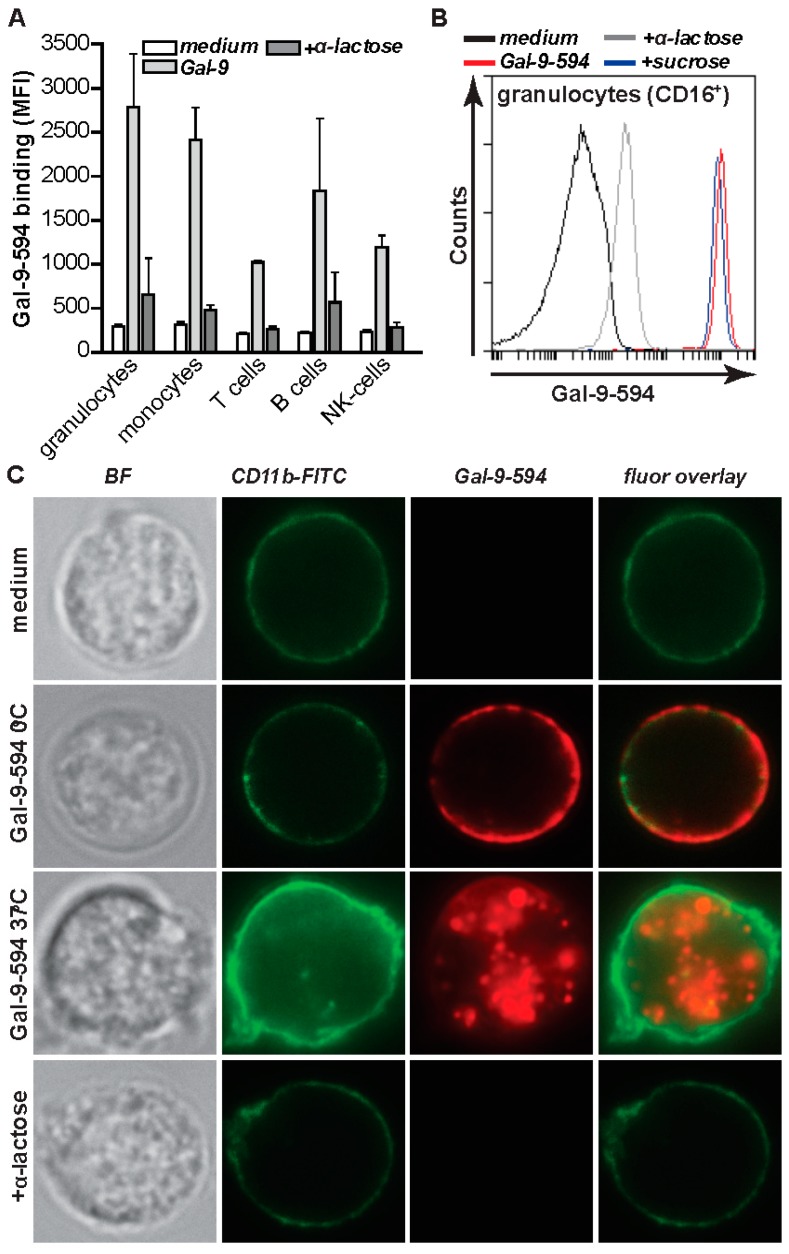
Gal-9 binds to granulocytes and other leukocytes in a lectin-dependent manner. (**A**) Binding of Gal-9-594 to peripheral blood leukocyte populations (*n* = 2). Different populations were distinguished based on FSC/SCC and additional surface markers: CD16 (granulocytes), CD14 (monocytes), CD3 (T cells), CD20 (B cells), CD56 (NK cells). (**B**) Binding of Alexa-594-labelled Gal-9 (Gal-9-594) to granulocytes as determined by fluorescent microscopy (with CD11b-FITC co-staining) and flow cytometry in the presence or absence of competitive inhibitor α-lactose (40 mM) or the irrelevant carbohydrate sucrose. (**C**) Binding and internalization of Gal-9-594 (+/− α-lactose, 40 mM) to granulocytes (counterstained with CD11b-FITC) at 0 and 37 °C visualized by fluorescent microscopy (representative picture of two independent experiments). Mag. ×100; cell diameter ~12 μm.

The above results suggest that Gal-9 is upregulated in patients with RA, BR, and particularly BRRA, yielding serum levels of up to 8000 pg/mL (~240 pM). Further, Gal-9 was previously reported to be present in bronchioalveolar lavage (BAL) fluid of BR patients at a concentration up to 1000 pg/mL (~31 pM) [39,40]. Therefore, we next evaluated potential immunomodulatory effects of Gal-9 on granulocyte. First, binding of Gal-9 to leukocytes from HC were assessed using a fluorescently-labeled recombinant form of Gal-9 [41] (Figure 2A). Gal-9 strongly bound to HC granulocytes (Figure 2A). Binding by Gal-9 was inhibited by α-lactose, but not the irrelevant sugar sucrose, and therefore carbohydrate recognition domain (CRD)-dependent (Figure 2A,B). Gal-9 also bound to other peripheral blood immune cells, although highest level of binding was found on granulocytes (Figure 2A). Of note, granulocytes in this study minimally expressed T cell immunoglobulin and mucin domain-3 (TIM-3), which is a proposed receptor for Gal-9 on some various cell types [5] (Figure S2B). This indicates that in our experiments, TIM-3 is unlikely to be the predominant receptor for Gal-9, a finding in line with recent other reports that TIM-3 is not the only receptor for Gal-9 on immune cells [6,12,42]. Binding of Gal-9 to granulocytes at 0 °C clearly showed membrane staining of granulocytes, as defined by co-localization with the cell surface-associated integrin CD11b (Figure 2C). When incubated at 37 °C, surface binding was followed by Gal-9 internalization, which was blocked by α-lactose (Figure 2C). 

**Figure 3:**
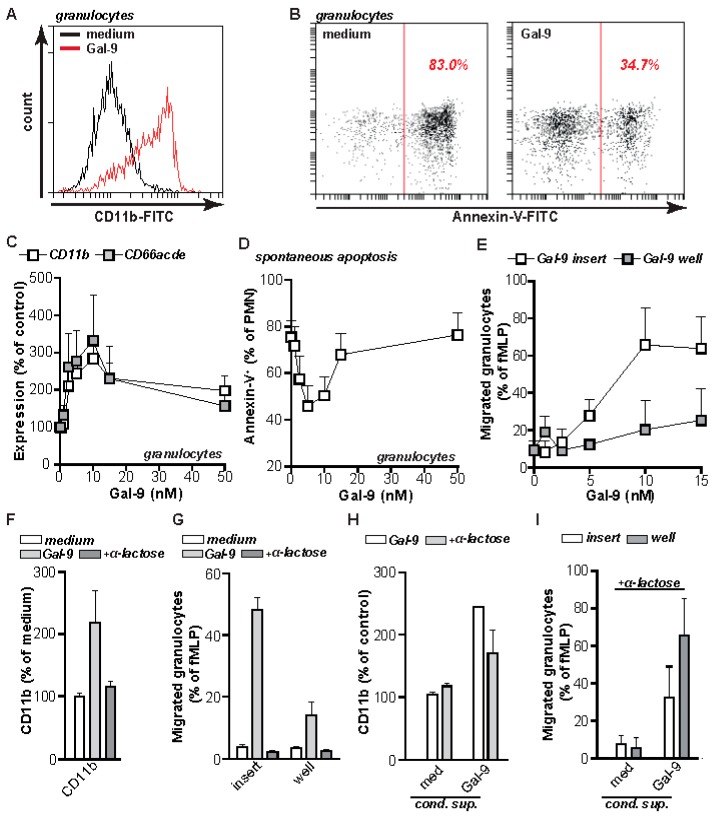
Gal-9 activates inflammatory functions in granulocytes. (**A**) Expression of CD11b on granulocytes after 16 h treatment with 15 nM Gal-9, determined by flow cytometry. (**B**) Granulocytes were treated with 15 nM Gal-9 for 16 h and analyzed for spontaneous apoptosis by measuring PS-exposure using Annexin-V-FITC. (**C**) Expression of CD11b and CD66acde on granulocytes after 16 h treatment with increasing concentrations of Gal-9, determined by flow cytometry. (**D**) Granulocytes were treated with increasing concentrations of Gal-9 for 16 h and analyzed for spontaneous apoptosis by measuring PS-exposure using Annexin-V-FITC. (**E**) Granulocyte cell migration after 16 h of incubation with Gal-9 using a trans-well system. (**F**) Gal-9-induced CD11b expression the presence of α-lactose. (**G**) Gal-9-induced granulocyte migration in the presence of α-lactose (40 mM). (**H**) As in (**A**), using Gal-9 or conditioned supernatant of Gal-9 treated leukocytes +/− α-lactose (40 mM) to inhibit direct Gal-9-mediated effects. (**I**) As in (**E**), using Gal-9 or conditioned supernatant of Gal-9 treated leukocytes in the presence of α-lactose (40 mM) to inhibit direct Gal-9-mediated effects. All experiments were at least performed in triplicate with granulocytes from independent donors.

Interestingly, the intensity of the CD11b staining increased upon incubation with Gal-9 at 37 °C, which indicates that treatment of granulocyte with Gal-9 triggers activation (Figure 2C). Indeed, expression of CD11b increased upon Gal-9 treatment as measured by flow cytometry (Figure 3A). 

In line with granulocyte activation, Gal-9 increased longevity of granulocytes with Gal-9 treatment reducing spontaneous granulocyte apoptosis after 20 h (Figure 3B). Both granulocyte activation, determined by CD11b as well as CD66acde expression, as well as granulocyte longevity dose-dependently increased upon treatment with Gal-9 (Figure 3C,D). To further evaluate the effect on granulocyte activation of Gal-9, granulocyte migration assays were performed in a trans-well system. Addition of Gal-9 to the insert induced migration of granulocytes, whereas addition of Gal-9 to the well only minimally triggered migration of granulocytes (Figure 3E). This migration data suggests that Gal-9 is a direct activator rather than a chemo-attractant for granulocytes. Activation of granulocytes, based on CD11b upregulation as well as migration, was dependent on CRD specific binding, as Gal-9 effects were inhibited by co-treatment with α-lactose (Figure 3F,G). In addition, upregulation of CD11b was also induced when supernatant of Gal-9-treated granulocytes was added to freshly isolated granulocytes (Figure 3H). Similarly, the addition of supernatant harvested from Gal-9-treated granulocytes to the insert or well of a trans-well system induced migration of granulocytes (Figure 3I). In contrast to direct addition of Gal-9 to granulocytes, the addition of supernatant to the well induced more migration of granulocytes than when added into the insert (compare Figure 3E,I). Importantly, the addition of α-lactose to the conditioned supernatant did not abrogate CD11b upregulation, nor migration of granulocytes (Figure 3H,I). Taken together, this data suggest that the effects induced by conditioned supernatant are not a direct effect of Gal-9, but rather caused by pro-inflammatory molecules, such as cytokines, induced by treatment of granulocytes with Gal-9.

### 2.3. Gal-9 Activates Pro-Inflammatory Cytokines Leading to Intercellular Adhesion Molecule 1 (ICAM-1) Expression

**Figure 4:**
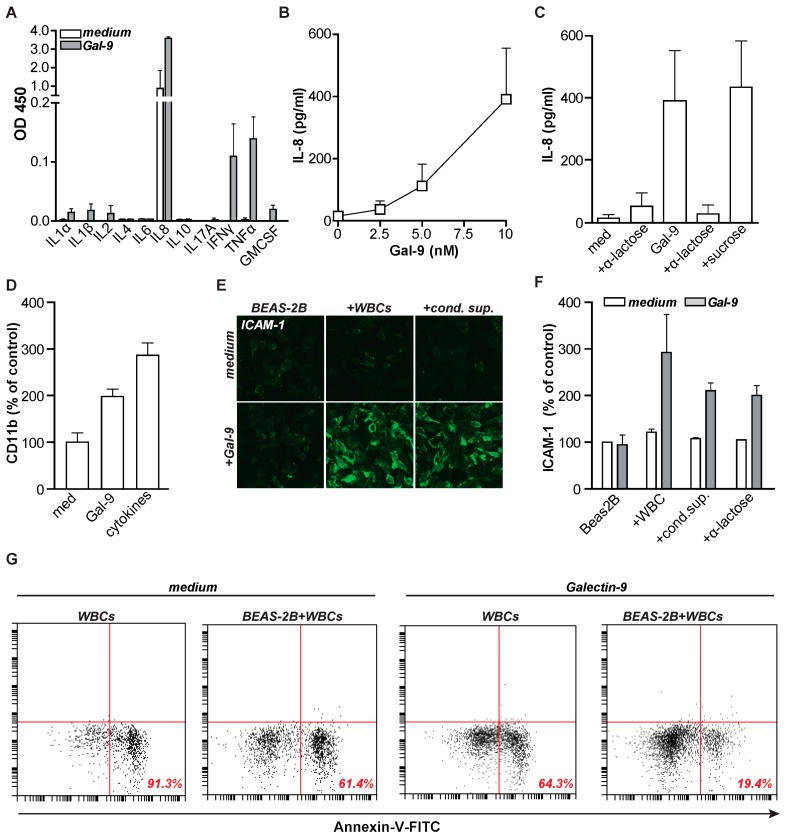
Gal-9 induces pro-inflammatory cytokines which induce Intercellular adhesion molecule 1 (ICAM-1) expression. (**A**) Screen for cytokines in supernatants of white blood cells (WBCs) after 16 h Gal-9 treatment (10 nM) using a multi-analyte ELISA. (**B**) IL-8/CXCXL8 ELISA in the supernatants of WBCs treated with increasing concentrations of Gal-9. (**C**) ELISA to determine IL-8 concentrations in the supernatants of WBCs treated with 10 nM Gal-9 +/− α-lactose (40 mM). (**D**) CD11b expression on granulocytes after 16 h Gal-9 treatment (10 nM) or a cytokine cocktail (400 pg/mL IL-8, 15 pg/mL TNF, 20 pg/mL IFN-γ) containing the cytokines that are also induced by Gal-9 treatment. (**E**) Fluorescent pictures of ICAM-1 expression on Beas-2B cells after incubation with WBCs +/− Gal-9 (10 nM) or conditioned supernatant harvested from medium- or Gal-9-treated WBCs. Magnification ×20 (**F**) As in (**E**), measuring ICAM-1 expression by flow cytometry. (**G**) Granulocyte viability measured by Annexin-V-FITC in the presence or absence of Beas-2B when treated with medium or Gal-9 (10 nM). All experiments were at least performed in triplicate.

To identify the potential induction of pro-inflammatory cytokines upon Gal-9 treatment, the cytokine profile in supernatant of Gal-9 treated leukocytes (containing all white blood cells), was determined using a multi-cytokine array (Figure 4A). 

Indeed, supernatant from Gal-9-treated leukocytes contained markedly elevated levels of interleukin-8 (IL-8) as well as elevated levels of interferon-gamma (IFN-γ), tumor necrosis factor (TNF), and a minor increase in granulocyte-macrophage colony-stimulating factor (GM-CSF). Further, quantitative analysis of cytokine levels demonstrated a dose-dependent induction of IL-8 from ~36 pg/mL at 2 nM Gal-9 to ~400 pg/mL at 10 nM Gal-9 (Figure 4B). Again, this induction of IL-8 secretion by Gal-9 was dependent on CRD interactions as it was blocked by α-lactose, but not sucrose (Figure 4C). Similarly, IFN-γ and TNF secretion dose-dependently increased upon treatment with Gal-9 (Figure S3A,B) and was blocked by α-lactose (Figure S3C). Of note, the level of secreted IL-8 upon treatment with Gal-9 correlated with the induction of migration of granulocytes (Figure S3D), which corresponds with the neutrophil chemotactic function of this cytokine.

To further confirm the importance of the pro-inflammatory cytokines that were secreted by leukocytes upon treatment with Gal-9, a cytokine cocktail containing the measured amounts of cytokines (400 pg/mL IL-8, 15 pg/mL TNF, 20 pg/mL IFN-γ) was added to freshly isolated granulocytes. As expected, both treatment with Gal-9 itself and treatment with the cytokine cocktail induced expression of CD11b (Figure 4D). Further, these cytokines and especially TNF are known to upregulate adhesion factors such as Intercellular adhesion molecule 1 (ICAM-1/CD54) and may thus contribute to Gal-9-induced tissue infiltration. Correspondingly, treatment of normal bronchial epithelial BEAS-2B cells with leukocytes and Gal-9, as well as conditioned supernatant of Gal-9-treated leukocytes, upregulated surface expression of ICAM-1 (Figure 4E,F). This further highlights the role of Gal-9 in neutrophil-mediated lung inflammation. In contrast, treatment of BEAS-2B with Gal-9 alone or with conditioned supernatant of untreated leukocytes did not impact on the expression of ICAM-1. Further, the addition of α-lactose did not block the effect of the conditioned supernatant, again suggesting the contribution of Gal-9-induced cytokines in this setting. Correspondingly, ICAM-1 expression was dose-dependently induced by TNF but was not further increased by the addition of IL-8 or IFNγ (Figure S3E). Such TNF-mediated induction of ICAM-1 expression on lung epithelial cells is in line with known literature [43,44].

### 2.4. Gal-9 Induces Anti-Microbial Inflammatory Responses In Vitro

**Figure 5:**
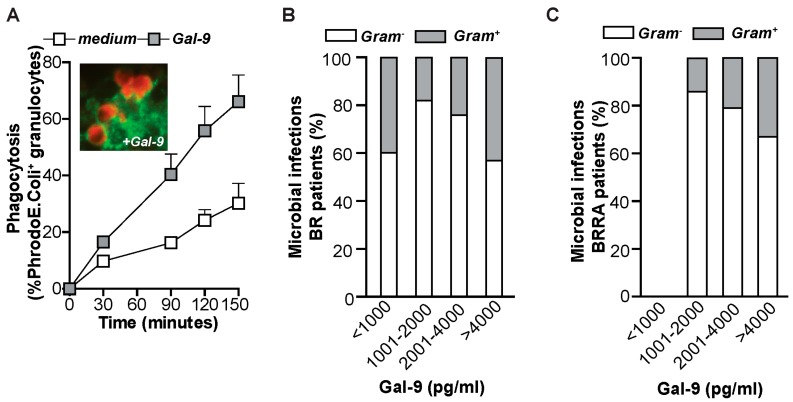
Gal-9 induces phagocytosis (**A**) Phagocytic uptake of PHrodo *E. coli* by granulocytes after pre-activation with Gal-9 (16 h, 10 nM). Inset: Fluorescent picture of phagocytosed *E. coli* (red) in granulocytes pre-treated with Gal-9 and stained for CD11b. 40× magnification. (**B**) Percentage of Gram-negative and Gram-positive lung infections occurring in BR and (**C**) BRRA patients with different Gal-9 serum levels.

CD11b and ICAM-1 are known interaction partners. Hence, the induction of both molecules by Gal-9 treatment may strengthen immune responses. Indeed, the treatment of leukocytes with Gal-9 in the presence of bronchial epithelial BEAS-2B cells further increased survival of granulocytes (Figure 4G), indicating that Gal-9 might regulate tissue infiltration of granulocytes. Of note, previous studies have implicated galectins in bacterial clearance. Therefore, we next assessed whether Gal-9 treatment of granulocytes could enhance uptake of bacteria. Indeed, treatment of granulocytes with Gal-9 enhanced granulocyte-mediated phagocytosis of rhodamine-labeled Gram-negative Escherichia coli (*E. coli)* by ~30% (Figure 5A). When performing this phagocytosis assay with fluorescently labeled Gal-9, the internalized Gal-9 did not co-localize with internalized/phagocytosed *E. coli* (Figure S3G). This lack of co-localization suggests that direct binding of Gal-9 to the bacteria is not the inducer of phagocytosis, but that phagocytosis is rather induced by Gal-9-mediated activation of granulocytes. Interestingly, elevated serum levels of Gal-9 also associated with a reduced percentage of Gram-negative bacterial infections in the small cohort of BR (Figure 5B) and BRRA patients (Figure 5C). Interestingly, the variety of microorganisms identified in BR and BRRA patient sputum varied with Gal-9 concentrations (Figure S4A–D). The diversity of pathogen in BBRA patients diminished in patients with Gal-9 serum levels exceeding 4000 pg/mL (Figure S4B,D). 

### 2.5. Gal-9 Induces Autoimmune Inflammatory Responses In Vitro

**Figure 6:**
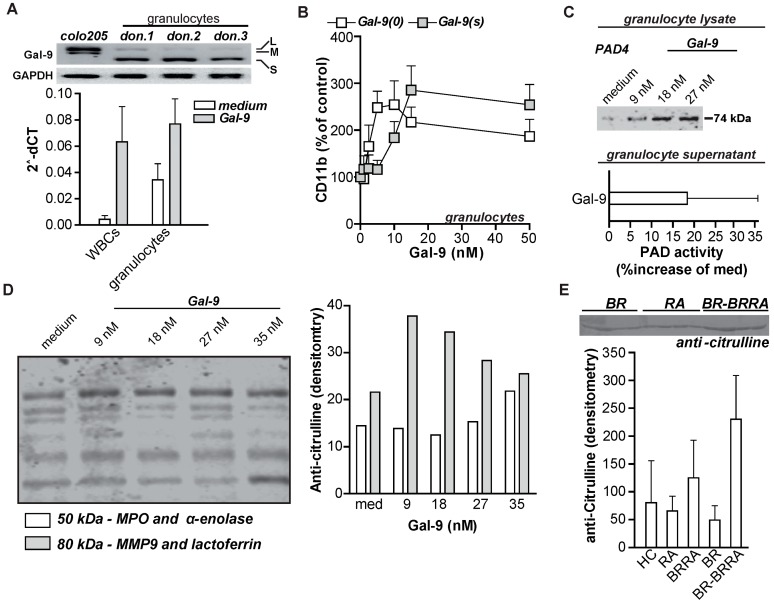
Gal-9 induces potentially autoimmune promoting responses in vitro. (**A**) Upper panel: detection of different Gal-9 isoforms as expressed by granulocytes using conventional PCR and subsequent gel electrophoresis. The colorectal cancer cell line Colo205 was used as a control. Lower panel: RTqPCR expression analysis of Gal-9 mRNA in total WBC or isolated granulocyte populations after treatment with Gal-9 (6 h, 10 nM). (**B**) Expression of CD11b on granulocytes after treatment with Gal-9(0) or Gal-9(s). (**C**) Western blot of protein arginine deiminase 4 (PAD-4) in cell lysates of granulocytes treated for 16 h with different concentrations of Gal-9. Lower panel: PAD-4 activity in supernatants of granulocytes treated with Gal-9 (16 h, 27 nM). (**D**) Detection of citrullination of intracellular proteins in granulocytes treated with Gal-9 (0–35 nM). Identification of citrullinated peptides was determined by LTQ-Orbitrap Velos mass spectrometry. (**E**) Relative differences of citrullination from 1 µL aliquots of serum proteins immunoblotted from HC, BR, BRRA, and BR patients that seroconverted to BRRA within 1 year (termed BR-BRRA). Immunoblots are representative of two or more experiments and bands from replicate gels were analyzed by mass spectrometry. All other experiments were performed at least in triplicate, except for the data in Figure 6A, which was in duplicate.

From the data outlined above, it is clear that sera levels of Gal-9 are elevated in RA patients with and without lung disease and that treatment with a recombinant stabilized form of Gal-9 can activate granulocytes in vitro. To further investigate the physiological relevance of this, we analyzed whether granulocytes also express endogenous Gal-9 and potentially upregulate Gal-9 expression upon activation. Of note, Gal-9 can physiologically occur as three different isotypes that only differ in their inter-domain linker length, i.e., Gal-9(M)/short linker, Gal-9(M)/medium linker and Gal-9(L)/long linker [2]. PCR analysis identified that granulocytes of three healthy control donors predominantly express mRNA for Gal-9(S) and only weakly express Gal-9(L), whereas control colorectal cancer cells express Gal-9(M) and (L) (Figure 6A). Interestingly, the treatment of leukocytes or isolated granulocytes further increased their Gal-9 expression as shown by RTqPCR (Figure 6A). As the predominant Gal-9 isoform expressed in granulocytes is Gal-9(S), the potential immunostimulatory effect of this isoform was evaluated. Treatment of granulocytes with Gal-9(S) induced almost identical activation of granulocytes as the recombinant stabilized form of Gal-9, with even higher levels of surface expression of CD11b at a concentration of 15 nM and above (Figure 6B). Similarly, both Gal-9(0) and Gal-9(S) upregulated the expression of CD66acde in granulocytes (Figure S3F). 

Interestingly, Gal-9 treatment of granulocytes also significantly increased the intracellular levels of the enzyme by protein arginine deiminase 4 (PAD-4) (Figure 6C). PAD enzyme catalyzes the citrullination of proteins, a post-translational modification that represents one of the most prominent and predictive pathogenic events for the development of RA. Corresponding to the elevated PAD-4 expression levels, there was an increase in PAD-4 activity detected in the supernatant of Gal-9 treated granulocytes (Figure 6C). In line with increased PAD-4 expression and activity, treatment of granulocytes with Gal-9 also dose-dependently increased the levels of citrullination of granulocyte intracellular proteins as determined by immunoblotting for citrullinated proteins (Figure 6D). Upon subsequent mass spectrometry analyses, two granulocyte intracellular proteins, namely matrix metalloproteinase 9 and myeloperoxidase, proved to be citrullinated upon Gal-9 treatment (Figure 6D). In line with these findings, an increase in citrullination of extracellular serum proteins was detected in BRRA patients and, most notably, in serum of BR patients that developed RA within the following 12–18 months (Figure 6E).

## 3. Discussion

**Figure 7:**
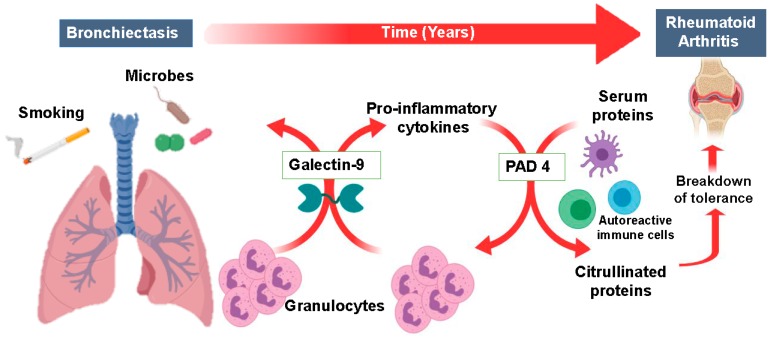
Galectin-9 is a possible promoter of immunopathology in rheumatoid arthritis by activating granulocytes. A possible scenario for the pathophysiological process of citrullinated peptide generation (via lung disease) through Gal-9 resulting in the development of RA. Following inflammation in the lung and infiltration of granulocytes, release of Gal-9 aids resolution of pro-inflammatory cells, leading to PAD-4 activation in granulocytes. Subsequently, this can lead to citrullination of intracellular proteins (as in vitro demonstrated in the current study) that upon release from cells can trigger and autoimmune response in immune cells leading to break down of tolerance over time.

In the current study we identified elevated levels of Gal-9 in serum of RA patients, which were even further increased in patients with BRRA. Further, we demonstrated that the in vitro treatment of human leukocytes with Gal-9 activates granulocytes, as characterized by increased cytokine secretion, migration, and survival. Importantly, Gal-9-treated granulocytes increased the expression of PAD-4, resulting in citrullination of intracellular granulocyte proteins that are known contributors to RA pathogenesis. Therefore, we propose that Gal-9 is a possible promoter of immunopathology in rheumatoid arthritis through its stimulatory effect on granulocytes (Figure 7).

The detection of elevated serum levels of Gal-9 in RA patients in this study is in line with a previous study that showed elevated levels of Gal-9 in synovial fluid samples of RA patients [45]. Here, Gal-9 induced apoptosis of synovial fibroblast in vitro and in vivo, thereby inhibiting autoimmune arthritis. Gal-9 levels may therefore be elevated to help to counteract the disease. However, the authors also state that Gal-9 may be involved in the development of RA, for which we provide evidence in our current study using an extensive set of in vitro analyses. Specifically, Gal-9 had a pro-inflammatory effect on granulocytes by reducing spontaneous apoptosis, inducing migration and triggering the secretion of the pro-inflammatory cytokines IL-8 and TNF. Furthermore, the treatment of granulocytes with Gal-9 up-regulated the expression and activity of PAD-4, a key enzyme that citrullinates proteins, which is also increased in bronchial lavage of RA patients [32]. Importantly, anti-citrullinated protein/peptide antibodies (ACPAs) are a hallmark of RA [46], and their presence can predict the development of RA even before clinical manifestation of the disease [47,48]. The precise mechanism of PAD-4 activation and increased protein citrullination by Gal-9 is unknown, but may be triggered by the reported capacity of Gal-9 to increase and mobilize cytosolic calcium concentrations [49], which in turn can activate PADs [50]. Thus, the elevated Gal-9 levels in RA patients may contribute to PAD-4 activation, protein citrullination and subsequent generation of ACPAs, leading to the break-down of tolerance and development of RA (Figure 7).

Serum Gal-9 levels were even higher in BR patients and especially in patients with RA and BR (BRRA). Similarly, a previous study showed significantly higher Gal-9 levels in BALF of BR patients compared to healthy controls [39]. In addition, Gal-9 levels have been reported to be increased in other inflammatory diseases [40], and especially in diseases involving lung infections, including pneumonia [51,52], acute lung injury caused by malaria infection [25], respiratory tularemia [24] or, dengue virus [53], and extrapulmonary tuberculosis [54]. Thus, increased Gal-9 levels in serum or lung fluid are generally found in diseases involving the infected lung. Of note, we have previously shown that chronic bacterial lung infections as seen in patients with BR can lead to the development of RA [37]. As Gal-9 levels are higher in patients with lung infections, Gal-9 may be a driving force for the conversion of BR to BRRA. Although it is currently unknown where the elevated levels of Gal-9 arise from, T cells can secrete Gal-9 upon activation [55,56]. In line with this data, we found that human leukocytes, including T cells, as well as isolated granulocytes, increased the expression of Gal-9 mRNA upon activation with Gal-9. Further, Gal-9 can be secreted by activated endothelium [57], human mesenchymal stromal cells [58], and intestinal epithelial cells [59,60]. Therefore, in the case of BR patients, Gal-9 may be secreted by activated immune cells or activated (lung) epithelium and endothelium. The increased local Gal-9 levels may further activate granulocytes, leading to the upregulation of CD11b and IL-8 secretion, as shown in our in vitro model. In addition, in our in vitro studies, bronchial epithelial BEAS-2B cells increased surface expression of the adhesion marker ICAM-1, upon treatment with Gal-9 in the presence of leukocytes. Importantly, CD11b (in the Mac-1 complex) and ICAM-1 or well-known interaction partners and Gal-9 may therefore stimulate granulocyte recruitment and infiltration to the lung [61]. When this process of Gal-9 secretion and granulocyte recruitment is continued in the lung, this results in the perpetuation of granulocyte-driven inflammation whereby Gal-9 functions as an ‘alarmin’ [23,24,62]. In line with this theory, Gal-9^−/−^ mice have reduced infiltration of CD11b^+^ granulocytes upon lung infection [24]. Further, it has been previously reported that Gal-9 induces the degranulation of granulocytes and increases their potential to phagocytose bacteria [23]. The latter is in line with our results, whereby treatment of leukocytes with Gal-9 enhanced the phagocytosis of *E. Coli* cells. Further, elevated serum levels of Gal-9 were associated with a reduced percentage of Gram-negative bacterial infections in our small cohort of BRRA and BR patients. Thus, bacterial infections as commonly seen in BR patients may lead to increased Gal-9 serum levels, causing a continuous inflammatory response in the lung, eventually leading to break-down of tolerance (Figure 7).

In line with a potential role in autoimmunity, the elevated Gal-9 serum levels in RA patients correlated with disease activity scores (DAS-28) and CRP, particularly in current and ex-smokers. In contrast, we did not observe a correlation between Gal-9 and disease activity in non-smoking RA patients. Of note, both never and ever-smoking BRRA patients had higher Gal-9 levels as compared to never smoking RA patients. Therefore, it is possible that the combination of high Gal-9 levels with smoking influences disease activity. Indeed, smoking also increases PAD levels in the lungs [63]. In addition, Gal-9 levels did not correlate with anti-CCP serum levels, which would have strengthened our hypothesis that high serum Gal-9 levels are causing PAD-4 activity and subsequent formation of anti-CCP antibodies. However, time delay between the increase in Gal-9 levels and the actual formation of anti-CPP can impact the correlation studies. In addition, the stability and retention of Gal-9 in serum compared to anti-CCP may also influence a direct correlation between both factors. However, in ROC curve analysis, BRRA serum Gal-9 concentrations had a better diagnostic performance than anti-CCP and rheumatoid factor for predicting RA. 

In conclusion, RA and BR patients had significantly higher serum levels of Gal-9 compared to HC subjects with even higher levels in patients with the overlap syndrome BRRA. Gal-9 directly promotes pro-inflammatory antibacterial immune responses and increased PAD-4 activity, which we suggest may represent a possible mechanism for breakdown of tolerance and development of RA in susceptible individuals. Based on our current data, we propose that Gal-9 is released during a normal immune response against bacteria, where it can contribute to bacterial clearance but at sustained levels in chronic infection leads to breakdown of tolerance (see Figure 7 for illustration). 

## 4. Patients, Materials, and Methods 

### 4.1. Patients and Control Subjects 

A total of 77 RA patients, of which 40 had comorbid Bronchiectasis (BR), 40 BR patients, and 28 age-sex matched control subjects were studied (Figure 1F). Patients and controls were selected as previously described [37]. All the RA patients fulfilled the American College of Rheumatology (ACR) 2010 classification criteria for RA [64]. Recruitment was completed over 12 months using identical methodology and reviewed by the same researcher (EP) with full ethical approval Research ethics committee reference 10/H0903/66 & integrated research application number 69084; 9 July 2014. All recruited bronchiectasis patients (age > 18 years) were under respiratory specialist care, and had high-resolution computed tomography (HRCT), evidence of proven symptomatic non–cystic fibrosis bronchiectasis, and a history of two or more respiratory infections per year. There is no recognized clinical severity index for bronchiectasis, although one has recently been proposed and evaluated, which recommends monitoring FEV1, bacterial colonization, HRCT, and quality of life [65]. In our study, we measured CRP, rheumatoid factor, FEV_1_, FEV_1_ % Pred or breathlessness score in our BR cohorts (BR and BRRA), as a means of monitoring disease severity. 

The gender and age distributions were similar between patient groups and healthy controls. All RA patients were chosen based on having no clinical or radiological evidence of any lung disease. Chest X-ray and lung function results were also reviewed for all RA patients and when they were performed, were within normal limits. Chest radiologists who performed HRCT were not involved in the study. Patients in the BR cohorts were receiving follow up care from a respiratory consultant. Patients with any other form of lung disease in addition to BR were excluded from the study. This included all those with established interstitial lung disease, asthma, or advanced emphysema. All BR patients underwent a musculoskeletal examination by a rheumatologist and were excluded if they had a history of inflammatory joint pain, inflammatory arthritis, or any synovitis. Rheumatoid factor (RF) was present in significantly greater numbers of BR/RA patients compared with patients with RA alone. 

Demographic details, together with the date of onset of symptoms and date of diagnosis for RA and BR by face-to-face assessment undertaken by one of us (EP). Current RA therapy and a detailed smoking history were also recorded. There was no significant difference in the proportion of ex/current smokers in the BRRA and RA cohorts selected (15 vs. 20) or never smokers in the BRRA and RA cohorts (25 vs. 15). A low mean number of pack years in the ex-smokers was observed for both groups. 

RA disease activity and severity measures were recorded for both groups including the Disease Activity Score in 28 joints (DAS-28) performed by a single rheumatologist (EP), C-reactive protein (CRP) levels, radiological evidence of erosive disease, and RA autoantibody status. RA remission was defined by a DAS-28-CRP score < 2.6. Anti-CCP was measured in serum by enzyme linked immunosorbent assay (ELISA) using the Phadia Elia^TM^ (Phadia AB, Sweden—second generation assay). We classified levels of <7 U/mL as negative, levels of 7–10 U/mL as equivocal, and levels >10 U/mL as positive. Results greater than 30 U/mL (three-fold the laboratory upper limit of normal) were considered high positive. 

The percentage of BRRA and RA patients on DMARD alone was 45% and 50% respectively. The percentage of BRRA and RA patients on no DMARD/biological therapy was 13% and 5%, while 21% and 37% of BRRA and RA patients were on combined DMARD/biological medication. While 21% and 8% of BRRA and RA patients were on a biologic alone therapy. In addition, 10% of BRRA and 3% of RA patients were also prescribed oral prednisolone. 

### 4.2. Serology 

Serum anti-CCP and rheumatoid factor was measured as described [36]. Anti-CCP levels of <7 U/mL were classified as negative, levels of 7–10 U/mL as equivocal, and levels >10 U/mL as positive. Rheumatoid factor levels <14 U/mL were classified as negative, levels >14 U/mL as positive. Galectin-9 serum concentration was measured using ‘Quantikine’ ELISA (R&D Systems, Abingdon, UK). Demographic parameters including RA therapy, detailed smoking history, date of onset of symptoms, date of diagnosis for RA and/or BR by face-to-face assessment, Disease Activity Score in 28 joints (DAS-28), C-reactive protein (CRP) levels, radiological evidence of erosive disease, and RA autoantibody status was recorded/collected by a single rheumatologist (EP). 

### 4.3. Isolation of Human Leukocytes 

Total leukocytes/white blood cell (WBC) populations were isolated from peripheral blood of consenting healthy subjects by ammonium chloride lysis. Granulocytes were isolated from peripheral blood by performing lymphoprep before ammonium chloride lysis or via a previously described protocol [66]. Granulocyte purity was confirmed using a hematology automated analyzer (Sysmex, Etten-Leur, The Netherlands) and Diff Quick staining [67]. 

### 4.4. Galectin-9 (Gal-9) 

Recombinant Gal-9 with a 2 amino acid inter-domain linker (termed Gal-9 throughout the manuscript) and the physiological short isoform of Gal-9 (Gal-9(S)) were produced as described previously [41].

### 4.5. Gal-9 Binding Assays

WBCs (3 × 10^5^ cells) were stained with Gal-9 conjugated to DyLight^®^ 594 (Gal-9-594) NHS (Piercenet, Thermo scientific, Breda, The Netherlands) for 1 h at 4 °C and washed with PBS to remove excess of Gal-9-594. Subsequently, Gal-9-594 labelled cells were divided over six different tubes and stained for 1 h at 4 °C with anti-CD16-PE, anti-CD14-PE, anti-CD3-PE, anti-CD20-PE, or anti-CD56-PE (Immunotools, Friesoythe, Germany) or non-marker control to identify cell populations. After washing with PBS, staining was analyzed using an Accuri C6 flow cytometer (BD Biosciences, Vianen, The Netherlands) and accessory CFlowPlus software.

### 4.6. Granulocyte Activation and Viability Assays

Total WBCs or isolated granulocytes (3 × 10^4^ cells) were incubated at 37 °C in 200 μL RPMI + 10% FCS for the indicated time points with/without of Gal-9 or Gal-9(S). After incubation, cells were stained by flow cytometry using CD11b-FITC, CD66acde-PE, or Annexin-V-FITC (Immunotools). In assays with conditioned supernatant, supernatants of treated WBCs were harvested and added to freshly isolated granulocytes with/without α-lactose or sucrose (40 mM, Sigma Aldrich, Zwijndrecht, The Netherlands) for 16 h at 37 °C.

### 4.7. ICAM-1 Expression Assays

BEAS-2B cells (human normal bronchial epithelium) were cultured on collagen coated plates in RPMI + 10% FCS for 72 h until a complete monolayer was formed. Subsequently, WBCs were added (3 × 10^4^ cells/condition) with/without 10 nM Gal-9 and lactose or sucrose (40 mM) and incubated for 16 h at 37 °C. Non-adherent WBCs were removed by three HBSS washes, after which ICAM-1 expression was visualized by fluorescent microscopy (Leica DMI6000B microscope and FDC365 FX camera, Newcastle, UK) after staining with anti-ICAM-1 (Immunotools) and secondary Goat-anti-mouse-Alexa-488.

### 4.8. Cell Migration Assays 

WBCs (1 × 10^5^ cells/condition) were placed in the upper chamber of a 24 trans-well system (3 µm pore size, ThinCertsTM Greiner Bio-one). Gal-9 (10 nM) or FMLP (100 nM; Sigma Aldrich) were added to the lower or upper chamber in RPMI + 10% FCS. For conditioned WBC supernatant, supernatant with α-lactose (to inhibit residual direct Gal-9 effects) was added to the lower chamber or fresh WBCs were resuspended in supernatant in the upper chamber. After 24 h, cell migration was determined by quantifying the number of cells in the lower chamber. Granulocyte migration was depicted as percentage of maximum migration as induced by FMLP added to the lower chamber.

### 4.9. Phagocytosis Assays 

WBCs (3 × 10^4^ in 200 μL) were incubated for 16 h at 37 °C with or without 10 nM Gal-9. Subsequently, 5 μL of pHrodo^®^ Red *E. coli* BioParticles^®^ (Life technologies, Bleiswijk, The Netherlands) were added. Phagocytosis was analyzed at indicated time points by flow cytometry or using fluorescent microscopy (40× magnification).

### 4.10. ELISAs on Granulocyte Supernatants

Supernatants were obtained following as described for granulocyte activation assays and analyzed using the Human Inflammatory Cytokines Multi-Analyte ELISArray™ Kit (Qiagen, MEH004A, Venlo, The Netherlands) and additionally with human IFNγ and TNF-α ready-SET-Go ELISAs (Ebioscience, 88-7316-86, 88-7346-86, Vienna, Austria) and CXCL8/IL-8 DuoSet ELISA (R&D Systems, DY208, Abingdon, UK).

### 4.11. RTqPCR, PCR, and Gel Electrophoresis

Granulocytes (6 × 10^6^) in RPMI + 10%FCS were treated with 10 nM Gal-9 for 6 h at 37 °C. RNA was isolated using Trizol^®^ isolation protocol (Invitrogen, Bleiswijk, The Netherlands) and quantified by Nanodrop-1000 (Nanodrop Technologies, Breda, The Netherlands). Subsequently, cDNA was obtained using Iscript cDNA synthesis kit (Bio-Rad, Lunteren, The Netherlands). cDNA targets were quantified with TaqMan gene expression assay using iTaq Universal SYBR Green Supermix (Bio-Rad) and commercial primer/probe sets for Gal-9 (Hs01088492m1; Invitrogen Life Technologies, Bleiswijk, The Netherlands) and the Taqman ABI 7900HT Sequence Detection System (Applied Biosystems, Foster City, CA, USA). All samples were run in duplicate (CT-value, SD < 1) and values were corrected for the expression of housekeeping genes B2M (Hs00984230_m1) and PPIA (Hs04194521_s1). To determine the expression of different Gal-9 isoforms, Gal-9 cDNA was amplified by 35 amplification cycles (1 min 95 °C, 1 min 60 °C, 30 sec 72 °C) using previously published primers [55]. Isoforms were visualized by UV-pictures of PCR products run on a 2% agarose gel + SYBR-SAVE (90 min, 200 V).

### 4.12. PAD-4 and Citrulline Western Blots and Mass Spectrometry 

Granulocytes (1 × 10^6^) were treated with Gal-9 (0–64.5nM final concentration) for 16 h. Granulocytes were pelted and resuspended in 100 µL lysis buffer (150 mM NaCl/1.0% NP-40/50 mM Tris, pH 8.0). Protein concentrations were determined by nanodrop spectroscopy and samples were adjusted to the same protein concentration. Then, 15 µL aliquots were run on Mini-PROTEAN^®^ 8–16% SDS-PAGE TGX 15-well gels (Bio-Rad). Gels were transferred onto nitrocellulose in a turboblotter (Bio-Rad). Blots were probed with 1 µg/mL rabbit anti-human-PAD-4 (Abnova PAB5507, Aachen, Germany), or 1 µg/mL rabbit anti-human citrulline (Abcam 100932, Cambridge, UK) washed three times in PBS-0.2% Tween-20 and once in PBS then incubated at RT for 1 h with a 1:15,000 dilution of IRDye 800CW goat anti-rabbit IgG (Li-Cor Biosciences 926-32211, Cambridge, UK). Blots were washed twice in PBS-0.2% Tween-20 and once in PBS alone and were analyzed on an Odyssey CLx Imager. Citrullinated protein bands from the gels were digested, fractionated, and analyzed using an LTQ-Orbitrap Velos mass spectrometer. 

### 4.13. PAD-4 Activity Assay

Granulocytes (1 × 10^6^ cells/mL) were incubated for 4–16 h at 37 °C in the presence of PMA (25 nM) or Gal-9 (27 nM). Supernatants were harvested and diluted 1:1 in deimination buffer enriched with additional CaCl_2_ (5 mM) and DTT (1 mM) to obtain a final concentration of 40 mM Tris-Hcl (pH 7.5), 5 mM CaCl_2_, and 1 mM DTT. PAD-4 activity was determined using the Modiquest PAD-4 enzyme assay (Modiquest Research, Oss, The Netherlands).

### 4.14. Statistical Analysis 

The Mann–Whitney nonparametric test (for unmatched groups) was used to compare differences between antibody responses in the cohorts of serum samples. Spearman’s nonparametric correlations between data sets were assessed. ROC calculations were performed using GraphPad 6 software. All cell biology experiments were performed on at least three separate occasions. 

## Supplementary Materials

Supplementary materials can be found at https://www.mdpi.com/1422-0067/20/16/4046/s1.
