# Supplementary figures and images for: Galectin-9 Is a Possible Promoter of Immunopathology in Rheumatoid Arthritis by Activation of Peptidyl Arginine Deiminase 4 (PAD-4) in Granulocytes
Source: Int J Mol Sci. 2019 Aug 19;20(16):4046. doi: 10.3390/ijms20164046 (PMC6721145; doi:10.3390/ijms20164046)

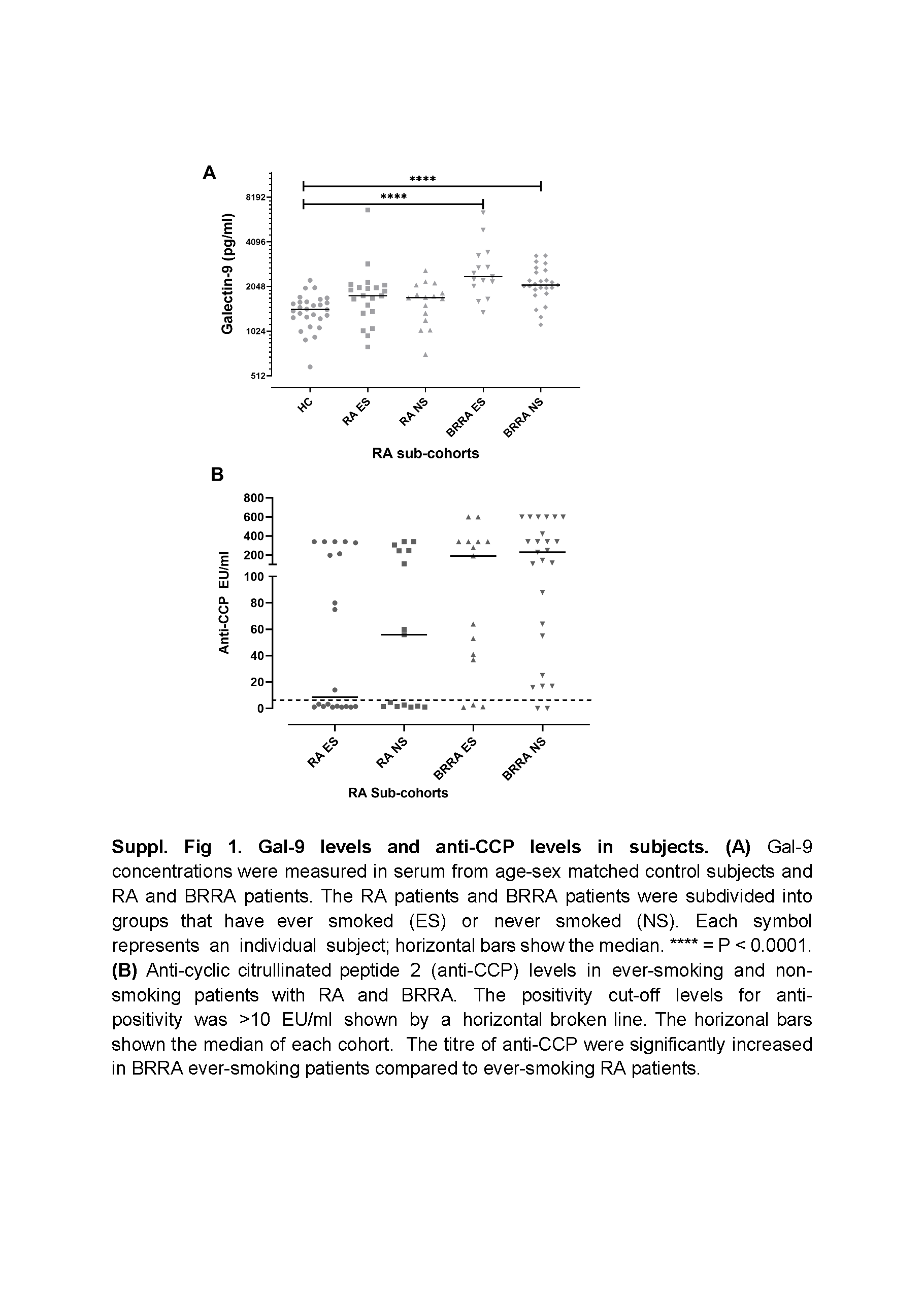

Supplement: Supplementary file 1 [file ijms-20-04046-s001.zip › New Suppl Fig 1 Gal and CCP levels.png]

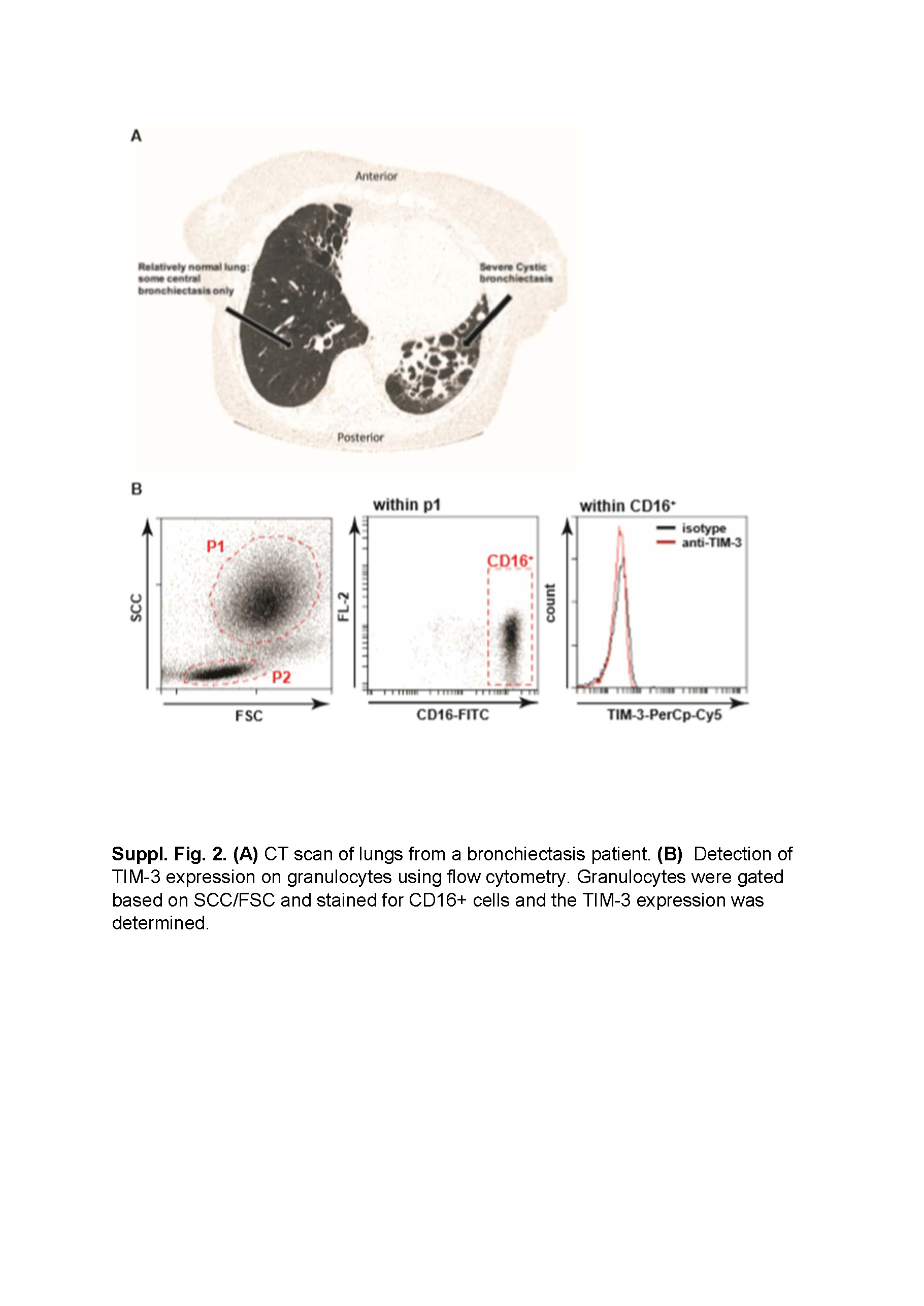

Supplement: Supplementary file 1 [file ijms-20-04046-s001.zip › New Suppl Fig 2.png]

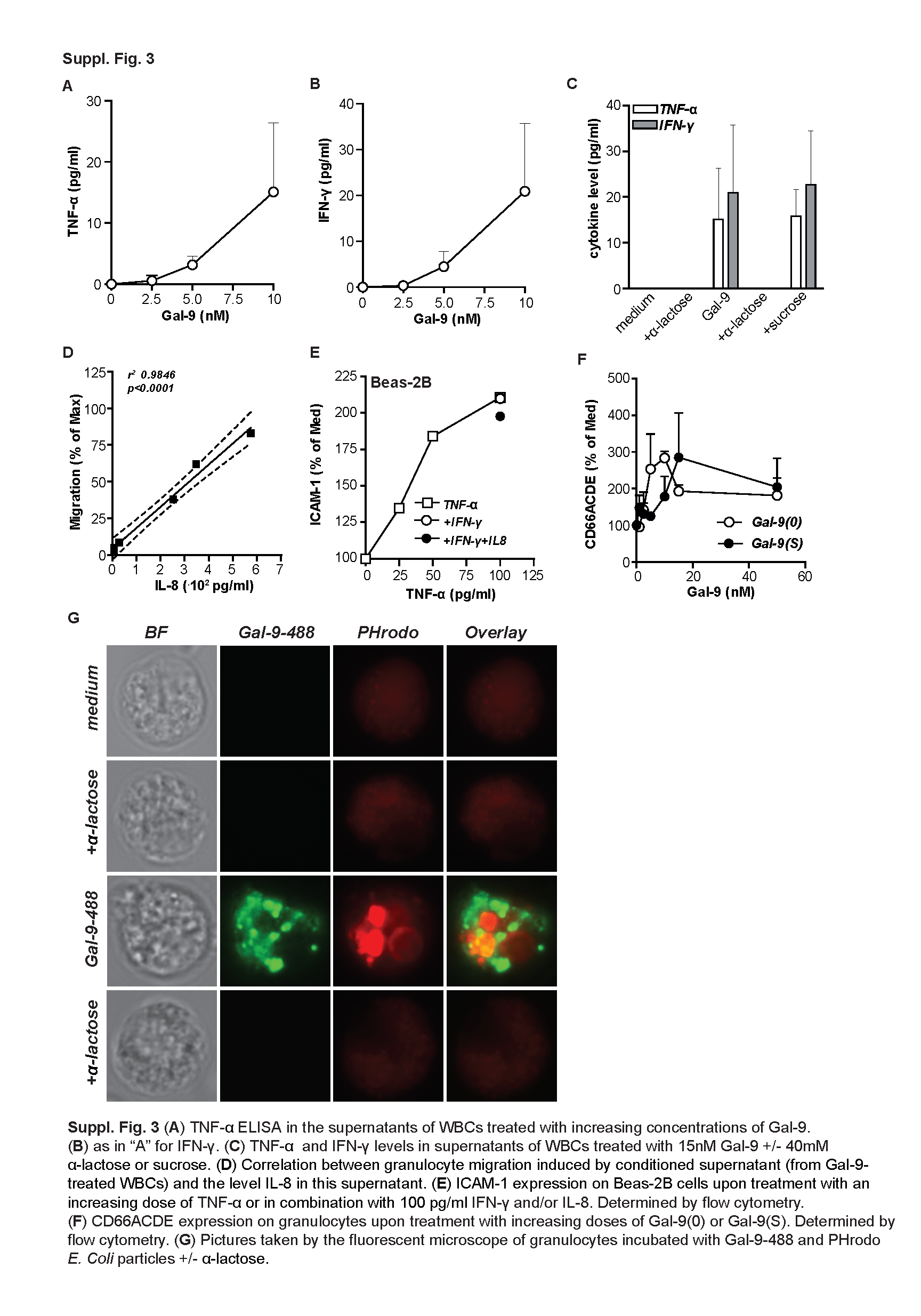

Supplement: Supplementary file 1 [file ijms-20-04046-s001.zip › New Suppl Fig3_Revision IJMS.png]

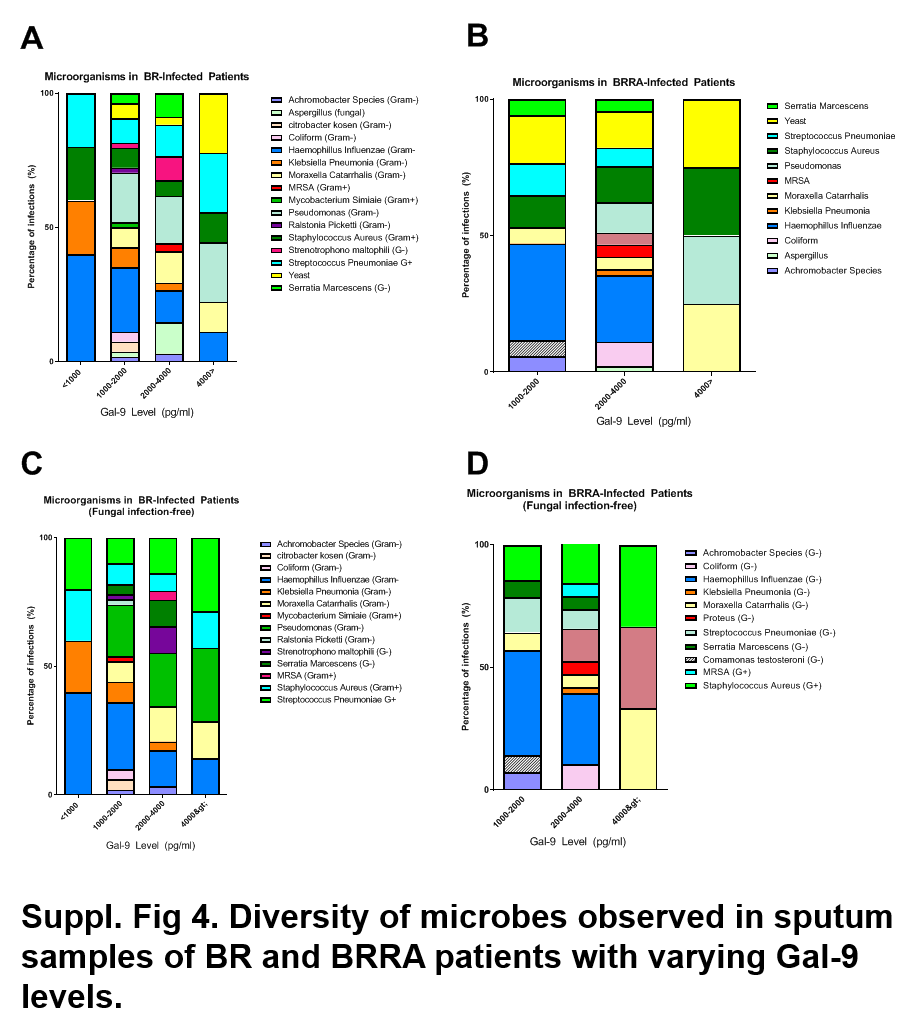

Supplement: Supplementary file 1 [file ijms-20-04046-s001.zip › New Supp Fig 4.png]
